# Clinical Quality Control of MRI Total Kidney Volume Measurements in Autosomal Dominant Polycystic Kidney Disease

**DOI:** 10.3390/tomography9040107

**Published:** 2023-07-12

**Authors:** Chenglin Zhu, Hreedi Dev, Arman Sharbatdaran, Xinzi He, Daniil Shimonov, James M. Chevalier, Jon D. Blumenfeld, Yi Wang, Kurt Teichman, George Shih, Akshay Goel, Martin R. Prince

**Affiliations:** 1 Meinig School of Biomedical Engineering, Cornell University, Ithaca, NY 14850, USA; 2 Department of Radiology, Weill Cornell Medicine, New York, NY 10065, USA; 3 Department of Medicine, Weill Cornell Medicine, New York, NY 10065, USA; 4 The Rogosin Institute, New York, NY 10021, USA; 5 Columbia College of Physicians and Surgeons, New York, NY 10032, USA

**Keywords:** ADPKD, total kidney volume, quality control, outlier analysis, MRI, steady state free precession, T2, T1

## Abstract

Total kidney volume measured on MRI is an important biomarker for assessing the progression of autosomal dominant polycystic kidney disease and response to treatment. However, we have noticed that there can be substantial differences in the kidney volume measurements obtained from the various pulse sequences commonly included in an MRI exam. Here we examine kidney volume measurement variability among five commonly acquired MRI pulse sequences in abdominal MRI exams in 105 patients with ADPKD. Right and left kidney volumes were independently measured by three expert observers using model-assisted segmentation for axial T2, coronal T2, axial single-shot fast spin echo (SSFP), coronal SSFP, and axial 3D T1 images obtained on a single MRI from ADPKD patients. Outlier measurements were analyzed for data acquisition errors. Most of the outlier values (88%) were due to breathing during scanning causing slice misregistration with gaps or duplication of imaging slices (*n* = 35), slice misregistration from using multiple breath holds during acquisition (*n* = 25), composing of two overlapping acquisitions (*n* = 17), or kidneys not entirely within the field of view (*n* = 4). After excluding outlier measurements, the coefficient of variation among the five measurements decreased from 4.6% pre to 3.2%. Compared to the average of all sequences without errors, TKV measured on axial and coronal T2 weighted imaging were 1.2% and 1.8% greater, axial SSFP was 0.4% greater, coronal SSFP was 1.7% lower and axial T1 was 1.5% lower than the mean, indicating intrinsic measurement biases related to the different MRI contrast mechanisms. In conclusion, MRI data acquisition errors are common but can be identified using outlier analysis and excluded to improve organ volume measurement consistency. Bias toward larger volume measurements on T2 sequences and smaller volumes on axial T1 sequences can also be mitigated by averaging data from all error-free sequences acquired.

## 1. Introduction

Autosomal dominant polycystic kidney disease (ADPKD) is an inherited kidney disease leading to end-stage kidney disease (ESKD) requiring dialysis or transplantation at a median age of approximately 60 years [1]. In ADPKD, progressive kidney enlargement from numerous cysts precedes the measurable decline in kidney function by decades because serum-creatinine-based kidney function biomarkers of glomerular filtration rate lack sensitivity, particularly during the early stages. The CRISP study found that total kidney volume (TKV) in ADPKD patients increased exponentially and was negatively correlated to GFR, as determined by iothalamate clearance [2]. The Mayo Imaging Classification (MIC) is a prognostic marker in typical ADPKD patients in which height-adjusted TKV (ht-TKV) and age at a single time point estimate the intrinsic rate of kidney growth [3]. Annual TKV follow-up is also widely used in ADPKD clinical trials to monitor intervention efficacy, as well as clinically to monitor each patient’s personalized disease progression [4].

The increasing use of TKV in clinical decision-making and clinical trials [5,6,7] raises questions about the repeatability and reproducibility of TKV measurements and which determinants of quality control are necessary to identify and correct volume measurement errors. CT and MRI both produce cross-sectional kidney images that can be manually contoured for organ volume measurements [8], but MR is commonly recommended because it avoids the risks of ionizing radiation, which can become considerable in patients having serial CT scans [9]. MR-based TKV, calculated by ellipsoidal estimation from length, width, and depth measurements, has limited repeatability [10,11]. Manually contouring the renal boundaries on every slice appears to have achieved high intra-observer repeatability with a percent difference of 0.8–3.5% and high inter-observer repeatability of 1.2–7.1% [10,11,12,13,14,15]. Moreover, several artificial intelligence (AI)—assisted segmentation algorithms showed high agreement with manual segmentation results with noninferior repeatability and substantial time savings [16,17]. However, this intra- and inter-observer repeatability may not translate directly into TKV measurement reproducibility in clinical practice because these authors fail to consider the variability introduced by different data acquisitions settings. Furthermore, TKV measurements in research are often single center data using one or two standardized imaging protocols (mostly coronal T2 or coronal T1) on a limited number of MRI scanners with experienced, expert observers making the measurements [10,11,12,13,14,15,18,19]. Thus, the generalization from these conclusions may be limited.

In contrast, routine clinical MRI for ADPKD patients includes multiple pulse sequences and imaging protocols, including T1, T2, and steady-state free precession sequences (SSFP) [20] in axial, coronal, and/or sagittal planes with a variety of imaging parameters. Unlike research protocols, where precise data acquisition protocols are strictly enforced, errors in image acquisition and subsequent segmentation are more likely in the clinical setting and less likely to be noticed and corrected. The large variability in liver and kidney size sometimes extending deep into the pelvis prompts technologists to adjust standardized protocols to obtain adequate anatomic coverage, further increasing the possibility of error. These errors may include: (1) image misregistration from multiple breath-holding acquisitions [19]; (2) kidney volume gain or loss due to breathing motion during scanning [19]; (3) segmentation errors, particularly hemorrhagic cysts or renal cysts bordering a polycystic liver [15]; (4) typographical errors in recording measurements; (5) kidneys not entirely included within the field of view or (6) artifacts that are not completely understood. Thus, contouring measurements of TKV in the clinical setting can produce erroneous results.

Recently, Dev et al. have proposed using deep learning for model-assisted segmentation of multiple pulse sequences of an MRI exam in order to utilize the power of averaging to reduce random measurement variation thereby improving reproducibility [16]. The purpose of this study is to determine how well TKV measurements agree among various MRI pulse sequences and to identify sources of error by identifying outlier measurements. By excluding errors discovered, the potential improvement in data consistency and TKV measurement reproducibility is determined.

## 2. Materials and Methods

### 2.1. Patients and Study Design

This HIPAA-compliant study was approved by the Weill Cornell Medicine Institutional Review Board. The retrospective review of existing medical images and patient data was considered minimal risk, and the requirement for informed consent was waived. Consecutive abdominal MRI exams (*n* = 130) from 20 January 2022–4 August 2022 in 122 ADPKD patients were included. Five MR pulse sequences included in the routine clinical protocol were analyzed to measure TKV, including axial and coronal single-shot fast spin echo (T2 or HASTE) T2-weighted, axial 3D spoiled gradient echo Dixon T1 (water), axial and coronal steady-state free precession (SSFP). MR exams missing one or more pulse sequences were excluded. 

### 2.2. MRI Acquisition

All MRI examinations were routine clinical abdominal MRIs without contrast performed on ADPKD patients using standardized protocols across seven MR scanners in imaging centers affiliated with Weill Cornell Medicine (Supplementary Table S2). Preferably, both kidneys and the liver were scanned within one acquisition for each sequence in a single breath hold. If more than one breath hold was necessary, the patient was given the same breath-holding instruction for each breath hold. When liver/kidneys were too large to be covered with a single acquisition, two acquisitions (upper and lower) for liver and kidneys were scanned separately and composed into a single stack of images (AdW Workstation: add/subtract feature GE Healthcare, Waukesha WI, USA); the patient was given the same breath-holding instructions for each breath hold.

### 2.3. Total Kidney Volume Measurements

Left and right kidneys were initially segmented using a 2D U-net deep learning algorithm described previously, separating the right and left kidney based upon image midline and updated by training on images from 397 patients including images from all five MRI pulse sequences [16,21]. However, after processing all five sequences on several studies, we determined that there were many outliers caused by segmentation errors related to stray labeled voxels in remote areas of the images (e.g., elbow, urinary bladder, stomach labeled as kidney) and errors in the separation of right and left kidneys when they were large and crossed the midline. Accordingly, we used the same training data to train a nnU-Net-based 3D deep learning algorithm calculating the centroid of the two largest segmented renal volumes to separate right from left kidney and eliminate stray labeling of voxels remote from kidneys. This revised model was applied to all images in the cohort. Model outputs were then corrected by three expert observers (CZ, AS, HD) all with experience manually contouring kidneys in over 100 cases using ITK-SNAP version 3.8.0 (Penn Image Computing and Science Laboratory, Philadelphia, PA, USA) [22] and 3D Slicer version 4.11 (www.slicer.org, Boston, MA, USA) [23]. Manual corrections were performed blinded to all clinical information. Left and right kidney volumes were calculated as the product of the total voxel count in the segmentation mask of each kidney, voxel in-plane area, and slice spacing. 

### 2.4. Acquisition Error Screening

A threshold value of 10% for the difference of inter-sequence TKV obtained by two different pulse sequences was used to screen for acquisition errors. Whenever the difference of TKVs obtained from two different sequences in one exam exceeded this threshold, a comprehensive review of the corresponding MRI images was conducted to identify four acquisition errors: (1) Breathing motion during scanning; (2) Different kidney breath-holding positions on acquisitions acquired with more than one breath hold; (3) Composing error from combining two overlapping acquisitions; (4) kidneys not entirely within the field of view. The problem was initially identified by one observer, followed by a review by two additional observers. In the event of a discrepancy, a consensus was reached by the three observers. We then determined a reference TKV for MRI exams by averaging the TKVs from error-free sequences or, if no sequence had an error, by averaging all five TKVs. This reference TKV was used as the gold standard TKV value.

### 2.5. Statistical Analysis 

All statistical analyses and quantitative summaries were performed using Python 3.7.9 (Python Software Foundation, Fredericksburg, TX, USA). The Shapiro–Wilk test was used to assess normality. Continuous variables were presented as mean ± standard deviation when normally distributed, or as median and interquartile range (IQR) when the distribution was not normal. The coefficient of variation and percent differences were utilized to evaluate intra-observer, inter-observer, and inter-sequence variation of TKV measurements. Additionally, a Bland-Altman analysis was conducted on the percent difference between TKVs obtained from different sequences to uncover any potential systematic bias from one pulse sequence relative to another in TKV measurement. The correlation was tested by Pearson correlation for linear relationships and Spearman correlation for monotonic relationships. The significance between the two groups was tested by Student’s *t*-test or Wilcoxon rank-sum test for normally and non-normally distributed data. For comparisons across multiple groups, a one-way analysis of variance was employed for normally distributed data, whereas the Kruskal-Wallis test was used for data not following a normal distribution. The *p*-values were adjusted using the Bonferroni correction to account for multiple comparisons. Dunn’s test was used as a post hoc analysis if the null hypothesis was rejected.

## 3. Results

### 3.1. Study Participants

**Figure 1:**
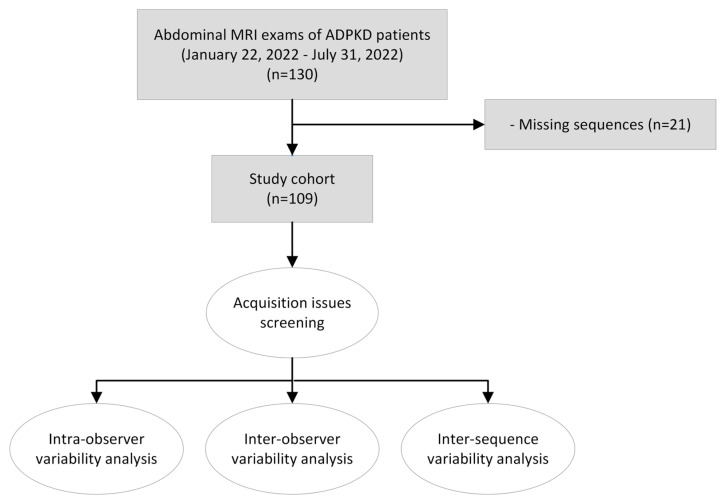
Study cohort flow chart.

After excluding MR exams missing one or more of the five studied sequences (*n* = 21), 109 MR exams from 105 patients were analyzed (Figure 1). 

Demographic, laboratory, and clinical details for the final 109 MR exams from 105 patients are provided in Table 1. The mean age of the study cohort was 46 ± 14 years, with slightly more females than males (ratio of female:male = 1.1:1). The most prevalent race was Caucasian (66%). The median TKV was 1279 mL (IQR: 761–2358 mL). The Mayo Classification was centered towards class 1B, 1C, and 1D. 

Weight, BMI, TKV, ht-TKV are reported as median [interquartile range]; all other variables are presented as mean ± standard deviation or counts (percentage). The TKV, ht-TKV, and Mayo class were calculated based on the average kidney volume of all five MRI pulse sequences from Observer 1 (CZ). BMI, body mass index; TKV, total kidney volume; ht-TKV, height-adjusted total kidney volume.

### 3.2. Inter-Observer Variations

Mean Total Kidney Volume (TKV) prior to correction of outliers for each sequence and the agreement among three observers (coefficient of variation, and maximum percent difference in TKV, reported as median (interquartile range) are shown in Table 2.

In assessing inter-observer variations in TKV measurements, we employed two metrics: (1) the correlation of variation in TKV measurements among three observers, and (2) the maximum percentage difference across all percentage differences of TKV between each pair of observers. Our findings revealed a low median coefficient of variation in TKV measurements among the three observers at 0.8% (IQR: 0.3–1.5%). Similarly, the median of the maximum percentage difference was also low at 0.9% (IQR: 0.4–1.7%). This indicates that our AI-assisted kidney segmentation generates reliable and reproducible TKV measurements among different readers.

Furthermore, we observed a moderate positive correlation between the coefficient of variation in TKV among the three readers and the absolute percentage difference between the model output TKV value and the mean TKV following manual adjustment Spearman correlation coefficient = 0.64, *p*-value < 0.0001) (Supplementary Figure S1). These findings suggest that inter-reader variation is inversely associated with AI segmentation model accuracy. The inter-reader variation metrics of TKV measurements were also calculated for each of the five MRI sequences and found to be consistently low across all sequences. However, the Kruskal-Wallis test revealed a statistically significant difference in the inter-reader variability of TKV measurements among the five sequences (*p*-value < 0.0001) (Supplementary Figure S2). Post-hoc Dunn’s test with Bonferroni correction for multiple comparisons showed that TKV measurements obtained from axial T1 and coronal T2 sequences had a significantly higher coefficient of variation compared to those obtained from axial SSFP, axial T2, and coronal SSFP sequences.

### 3.3. Inter-Sequences Variations 

Table 3 presents the variation in TKV measurements obtained from five different sequences for the model and each of the three readers. On average, among the three readers, each pair of measurements from two different pulse sequences was 5.4% different ranging from a minimum of 0.6% to a maximum of 12.4% with a mean CV of 4.6%. 

**Figure 2:**
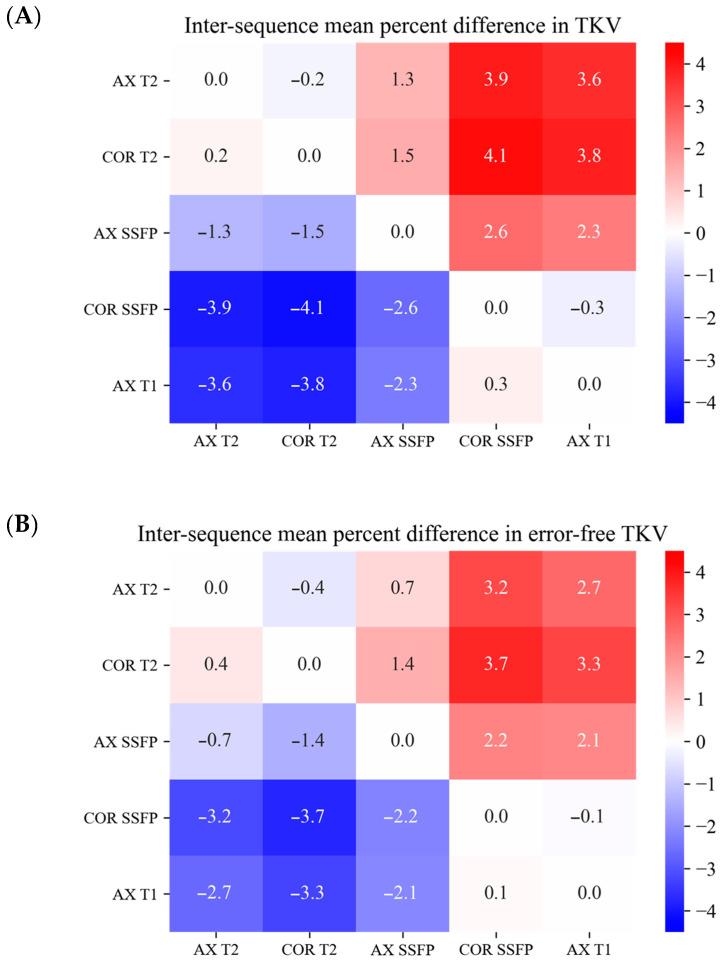
Heatmap showing mean percent differences in TKV between MRI sequences. (**A**) Among TKV obtained from all pulse sequences; (**B**) Among TKV obtained from error-free pulse sequences only (excluding 10% outlier values) Note the reduction in the intersequence mean percent differences post excluding outliers, particularly for the more extreme values which shows the benefit of excluding outlier measurements. AX: axial; COR: coronal.

Our heatmap showing mean percent differences in TKV between MRI sequences (Figure 2A) demonstrates that the T2 sequences, regardless of axial or coronal orientation, exhibited a bias toward larger TKV compared to the coronal SSFP and axial T1 sequences. The average percent difference ranged from 3.6% to 4.1%. In other words, the coronal SSFP and axial T1 sequences are biased toward smaller TKV measurements. The axial SSFP-derived TKV lies intermediate between these two groups.

### 3.4. Acquisition Errors

Out of 545 MRI pulse sequences drawn from 109 MRI examinations, 81 acquisition errors were identified by a 10% percent difference threshold (Table 4). These errors were found in 15% of all MRI pulse sequences and impacted 45% of the MRI exams. These errors may not be readily detected on images in the plane of acquisition. However, they could be detected by reviewing orthogonal reformations. 

**Figure 3:**
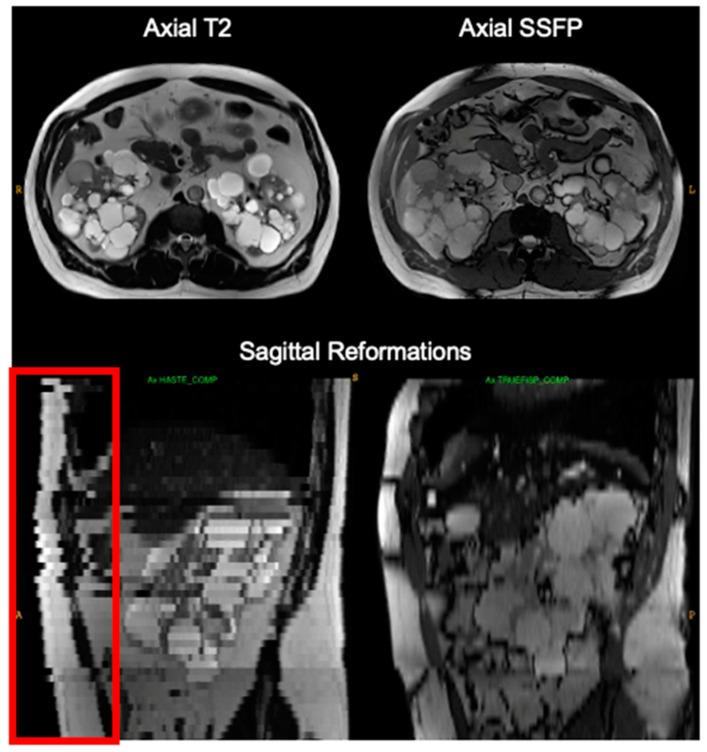
Breathing motion during scanning axial T2 (**left**) is not apparent on axial images in the plane of acquisition (**top left**) but is easily detected on sagittal (**lower left**) reformations with boundary discontinuities (red box). There was no breathing motion on the next pulse sequence, axial SSFP (**right**). This slice misregistration cannot be easily corrected but TKV measurement reproducibility is improved by excluding it from the TKV calculation.

Breathing during scanning caused slice misregistration evident on orthogonal reconstructions, e.g., on coronal reformations of axial acquisitions and vice versa (Figure 3). Duplicated or missing kidney slices but not necessarily bilaterally were identified affecting 35 pulse sequences (6% of total pulse sequences) and impacting 25% of exams. This higher-than-normal incidence of breathing motion errors during abdominal MRI acquisition is expected in ADPKD patients, given the limited lung expansion room due to increased kidney volume pushing up on the diaphragm and the extended scanning time needed to cover the enlarged liver/kidneys. These errors resulted in a median 7.8% difference from the reference TKV, ranging from −14% to 23%, and primarily affected T2 images (in 31 out of 35 cases). Specifically, breathing motion in axial T2 scans resulted in a TKV deviation from the reference TKV with a median of 9.3% and an interquartile range of 2.6% to 13%. For comparison, breathing motion in coronal T2 scans led to similar TKV deviation with a median of 7.8% and an interquartile range of 1.6% to 11%.

**Figure 4:**
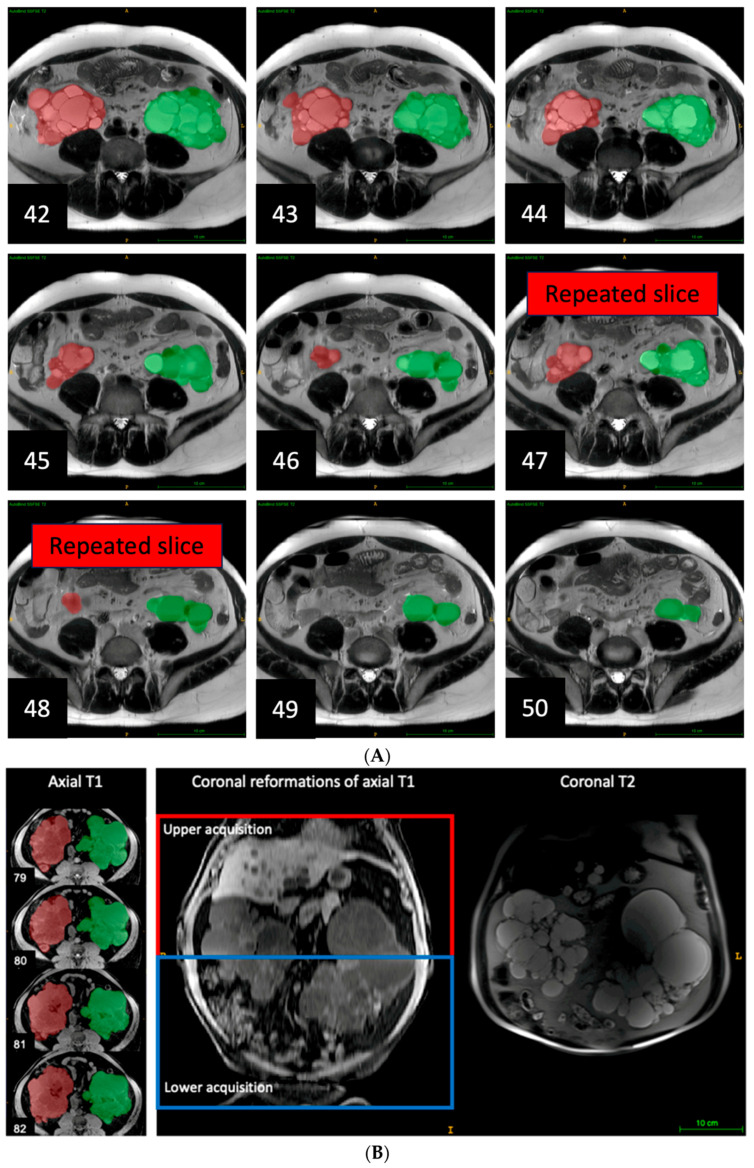
(**A**). Duplicated images due to acquiring images 1 to 46 during one breath hold and images 47 to 92 with another breath hold at a different respiratory position. This causes images 47 and 48 to overlap with images 45 and 46. This can be corrected by deleting images 47 and 48 which changes the TKV 3741 mL, pre-correction, to 3592 mL after correction; This 4.0% change brings the TKV measurement for this sequence more in line with TKV measured on the other sequences. Right kidney is labeled in red. Left kidney is labeled in green. (**B**). Missing images of axial T1 due to acquiring images 1 to 80 during one breath hold and images 81 to 160 with another breath hold at a different respiratory position. This causes a substantial change in kidney slice morphology between images 80 and 81 in axial T1, and a discontinuity in the coronal reformations of axial T1, which was not seen in the coronal T2. This error led to a 3.2% underestimation of TKV compared to the reference TKV.

Errors arising from using multiple breath holds at different breath-hold positions to complete an axial acquisition manifested as nearly duplicated or missing kidney slices, and uneven body boundaries on coronal, or sagittal reformations (Figure 4). Additionally, duplicated or missing kidney slices commonly occurred in the middle of the acquisition. This error was observed in 25 axial sequences, accounting for 5% of sequences and 19% of the exams causing a median TKV underestimation of 4.4% relative to the reference TKV, with percent differences ranging from −12% to 22%. Notably, axial T1 sequences represented nearly half of all pulse sequences with this error and mostly resulted in underestimation, with a median of −6.1% and an interquartile range of −7.4% to 4.4%.

**Figure 5:**
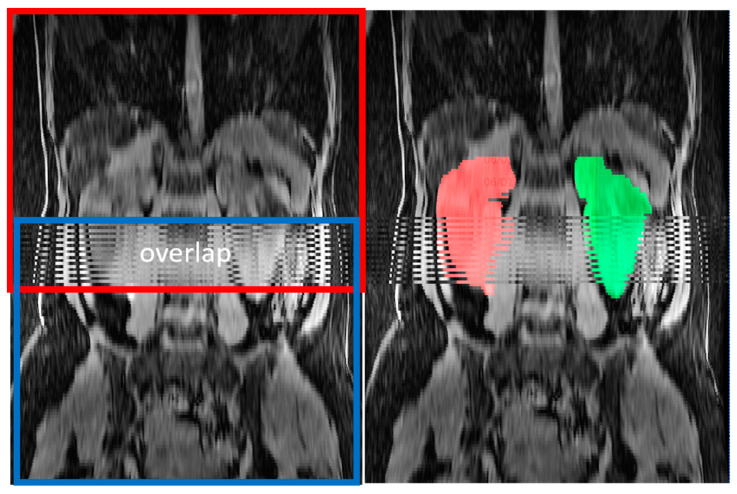
Composing errors from combining two, red and blue, overlapping acquisitions. In the region of overlap, the calculated organ volumes are erroneously doubled. This can be repaired by re-ordering the slice order and adjusting slice thickness based on the actually calculated spacing between slices. Right kidney is labeled in red. Left kidney is labeled in green.

Composing errors from overlapping acquisitions exhibited alternating strips (Figure 5) on orthogonal reformations. We identified composing errors in 17 exclusively axial MRI pulse sequences, including six axial T2 sequences, seven axial T1 sequences, and four axial SSFP sequences, representing 3% of pulse sequences and 11% of exams. These composing errors led to an overestimation of TKV relative to the reference TKV, with a median of 8.7% and a range from 2.5% to 29%.

**Figure 6:**
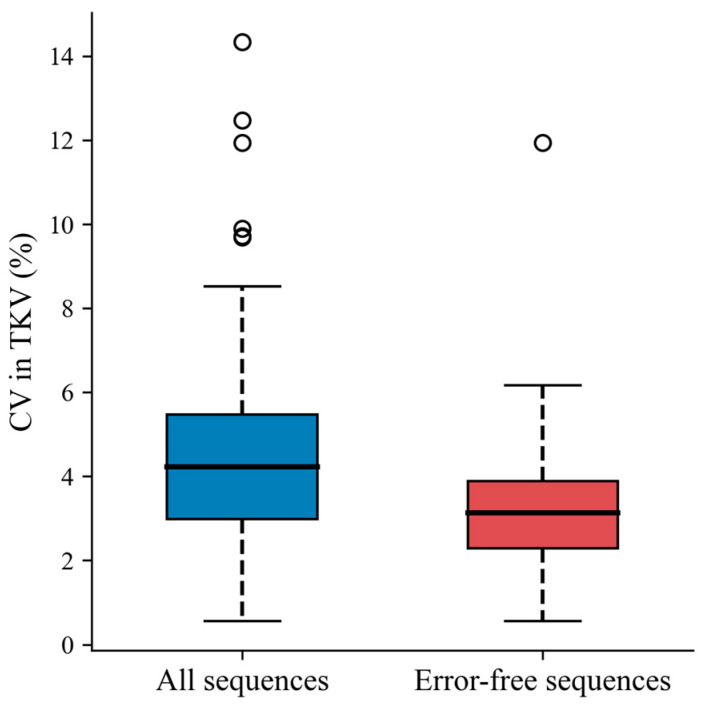
Boxplot of coefficient of variation of inter-sequence TKVs from all pulse sequences (median = 4.2%, IQR: 3.0–5.5%) versus error-free pulse sequences only (median = 3.1%, IQR: 2.3–3.9%, *p* < 0.0001). CV: coefficient of variation.

Incomplete kidneys (Figure 6), the fourth type of acquisition error, were identified exclusively in axial T1 sequences (*n* = 4) on 4% of exams. This error resulted in TKV underestimations of −7.8%, −8.2%, −8.7%, and 29% among the four affected sequences.

Axial T2, coronal T2, and axial T1 had the most acquisition errors, with 25, 19, and 24 sequences with errors detected out of 109 MR exams, respectively. In contrast, coronal SSFP had the fewest errors, with only 3 out of 109 exams affected. This may reflect the shorter scan duration for coronal SSFP which was thus more likely to be completed in a single breath hold. All coronal acquisitions (T2 and SSFP) in this study were affected exclusively by breathing motion, as they were single-acquisition and thus free from errors associated with multi-acquisitions. Moreover, axial T1 was devoid of breathing motion errors, as all slices in this 3D sequence were acquired simultaneously. 

After excluding sequences with acquisition errors, the average inter-sequence coefficient of variation among the five sequences reduced from 4.6% down to 3.2% (*p*-value < 0.0001, Figure 6). This demonstrates that the acquisition errors account, at least partially, for the significant inter-sequence TKV variations observed before the acquisition error screening.

We recalculated the sequence-to-sequence TKV percent difference matrix after excluding the sequences with errors, using the 10% percent difference threshold. A significant inter-sequence percent difference in TKV remained: the maximum percent difference was found where the TKV from coronal T2 was, on average, 3.7% larger than the TKV from coronal SSFP (Figure 2B). This remaining variation was partly explained by measurement bias.

**Figure 7:**
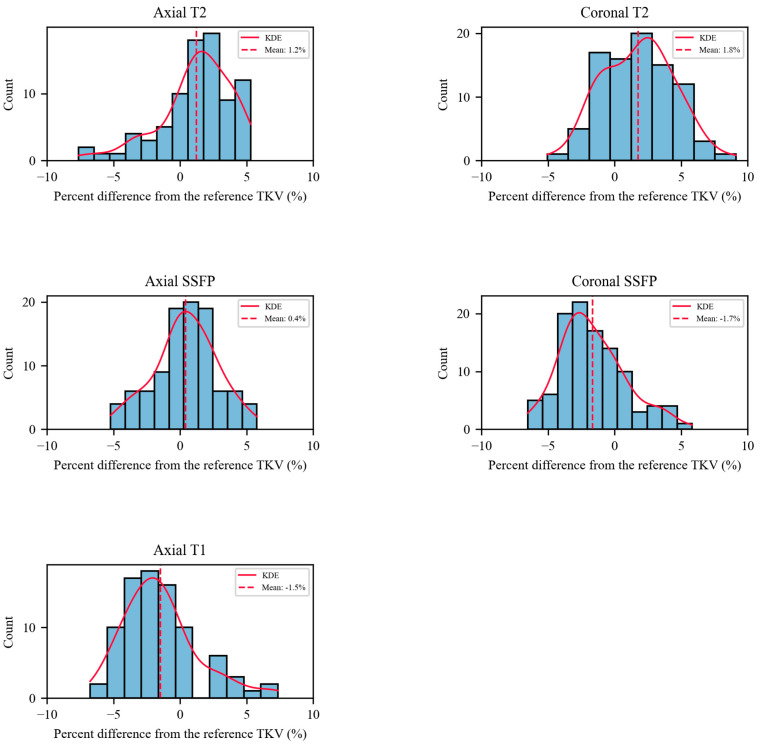
Histograms of the percent difference between the TKV obtained from each sequence and the reference TKV after excluding the sequences with acquisition errors. KDE: kernel density estimation.

Bias remaining in TKV measured from each sequence was assessed relative to the average of error-free sequences, the reference TKV. Relative to this reference TKV, both axial T2 and coronal T2 displayed an overestimation bias of 1.2% and 1.8%, respectively. Axial SSFP was closest to the reference with only a slight overestimation bias at 0.4%. On the other hand, coronal SSFP and axial T1 demonstrated underestimation bias of −1.7% and −1.5%, respectively (Figure 7).

## 4. Discussion

Total kidney volume measured on MRI is an important biomarker for ADPKD patients [24]. However, methods for quality control on this measurement have been lacking [10,14,16]. These data from 109 MR exams reveal multiple common MRI data acquisition errors that impact TKV accuracy and reproducibility including breathing during scanning causing slice misregistration, acquiring images with multiple breath holds at different respiratory positions, errors in composing two acquisitions together and kidneys not entirely within the field of view. It can be difficult to detect these errors which may not be readily apparent in their plane of acquisition. However, in this study, using model-assisted deep learning with three expert independent observers measuring TKV five times in each patient from five separate MRI pulse sequences, outlier analysis was able to identify aberrant measurements for a more thorough inspection. A low median inter-sequence agreement, 4.6% mean coefficient of variation, was reduced to 3.2% by excluding outlier measurements with errors. In addition, these data show a bias: T2 images overestimated TKV while axial T1 and coronal SSFP images underestimated TKV with a difference on the order of 3%. Finally, our AI model-assisted segmentations had improved inter-observer agreement (median coefficient of variation of 0.8%) compared to prior studies (a mean or median coefficient of variation of 1.7% to 7.1) [10,11,12,13,14,15,18,19].

Given the 2.8% to 5.5% average annual growth rate of TKV in ADPKD patients with or without tolvaptan treatment [25], the pulse sequence biases and data acquisition errors are important to address in order to have a consistent, meaningful TKV measurement for clinical decision making. If on one visit TKV is measured on T1 images and on the next visit the TKV is measured on T2 images there will be an approximately 3% apparent increase in TKV based just upon switching the sequence used for the measurement. The bias is potentially greater than the expected annual benefit from tolvaptan. Similarly, data acquisition errors resulted in differences in TKV measurements on an order of 5.0–8.5% on average, which exceeds the annual expected growth rate. 

Prior studies have recognized the potential negative impact of MRI acquisition composition and slice misregistration artifacts on TKV measurements. For example, Cohen et al. (2012) subjectively screened all axial reconstructed images of their patients (*n* = 17) for discontinuities from motion or misregistration [12]. However, prior studies have been limited. Stringent exclusion criteria often led to removing images with artifacts, or the focus was narrowed to specific MRI sequences less likely to exhibit these artifacts. Although this works for research studies, it is not practical for routine clinical imaging where it can be challenging to get patients to return for repeating an MRI that had artifacts. Bae et al. (2009) opted for breath-holding T1 over T2 imaging, because T2 was more prone to misregistration, motion artifacts, and heterogeneous tissue signal intensities, potentially leading to greater imprecision in kidney volume measurement [19]. However, in our experience, those advantages of T1 over T2 are offset by the more limited contrast of T1 which does not show the cystic kidneys as well, thereby making contouring the border more challenging. In addition, we found T1 had more composing errors, particularly in patients with enormous kidneys requiring multiple acquisitions.

We found axial images to be more prone to artifacts from the combination of multiple acquisitions, with both overestimation and underestimation of TKV. T2-weighted images acquired in 2D mode with single shot fast spin echo where each slice is acquired separately were more susceptible to breathing motion during acquisition. With 3D T1, images were all acquired simultaneously eliminating misregistration from breathing motion artifacts, but again it was more challenging to identify renal contours on T1. SSFP, a less commonly used sequence for TKV measurement compared to T1 or T2, was faster with a shorter scan duration requiring shorter breath holds and appeared to have less slice misregistration. Axial SSFP produced TKV measurements with the least systematic bias, differing by only 0.4% from the reference TKV. However, SSFP often had disturbing banding artifacts.

In addition to these technical issues, errors can also be introduced by human factors. For example, 4% of errors observed here were caused by the technologist failing to recognize that the kidneys were not entirely within the field of view. We also noticed that typographical errors could occur when kidney volume measurements are transcribed into the patient report. We eliminated typographical errors in this study by using a script to automatically extract kidney volume measurements into our research database, but for routine clinical reporting of kidney volume measurements, the possibility of transcription error should be considered. Finally, there is an error introduced by using a variety of MRI scanners with different software and calibrations [26]. Although this was not specifically investigated here, we noted the coefficient of intersequence variation varied from 3.5% to 5.2% among the seven types of MRI scanners used for acquiring images in this study (Supplementary Figure S5).

Given these findings, we advocate for a TKV measurement quality control process that includes measuring all clinically acquired routine MRI pulse sequences, particularly T1, T2, and SSFP sequences. Automatic segmentation algorithms that reduce radiologist segmentation time make this practical and fast. During scanning, MRI technologists should aim to acquire each imaging sequence in a single acquisition with a single breath hold as much as possible. Multiple acquisitions need to be planned carefully to ensure there is no overlap or gap between acquisition regions. Patients should be coached to maintain consistent breath-holding positions during multi-acquisition scans. The average, or weighted average depending on the patient’s characteristics, of TKV from all error-free acquisitions should be reported to mitigate the effect of pulse sequence bias. Ideally, this TKV measurement quality assurance process should take place automatically, while the patient is still on the scanner table so that MRI sequences with outlier values can be repeated immediately without having to call back the patient. Rapid quality assurance feedback to the MRI technologists will help them learn how to avoid data acquisition errors in the first place leading to accurate and reproducible TKV measurements for managing ADPKD.

In conclusion, MRI measurement of total kidney volume in ADPKD patients requires quality control to address data acquisition errors and mitigate pulse sequence biases.

## Figures and Tables

**Table 1:** Clinical and laboratory characteristics of the study cohort.

Study Cohort Number	109
Age at scan (years)	46 ± 14
Sex, male:female	51:58 (47% male)
Height (m)	1.7 ± 0.1
Weight (kg)	75 (62–86)
BMI	25 (22–28)
eGFR (mL/min per 1.73 m^2^)	69 ± 29
TKV (mL)	1279 (761–2358)
ht-TKV (mL/m)	782 (425–1383)
Mayo Class	
1A	10 (9%)
1B	29 (27%)
1C	36 (33%)
1D	20 (18%)
1E	14 (13%)
2 (atypical)	0 (0%)
Race	
Asian	14 (13%)
Black	2 (2%)
White	72 (66%)
Unknown	21 (19%)

**Table 2:** TKV measurement variation among three expert observers.

	Mean TKV (mL)					TKV Measurement Variation among Three Expert Observers	
Sequence	Model *	Reader 1 (CZ)	Reader 2 (AS)	Reader 3 (HD)	Average (%Δ from Mean) **	Coefficient of Variation (%)	Maximum Difference (%)
Axial T2	1910	1893	1905	1903	1900 (1.8%)	0.7 [0.3–1.3]	0.7 [0.3–1.4]
Coronal T2	1903	1880	1905	1909	1898 (1.7%)	1.2 [0.7–2.2]	1.3 [0.8–2.4]
Axial T1	1844	1853	1826	1824	1834 (−1.7%)	0.9 [0.5–2.0]	1.1 [0.6–2.2]
Axial SSFP	1872	1870	1875	1880	1875 (0.5%)	0.5 [0.2–1.2]	0.5 [0.2–1.3]
Coronal SSFP	1818	1819	1820	1822	1820 (−2.5%)	0.6 [0.3–1.5]	0.6 [0.3–1.6]
All sequences	1869	1863	1866	1868	1866 (0.0%)	0.8 [0.3–1.5]	0.9 [0.4–1.7]

* Model: the auto-segmentation algorithm; ** Average of the three readers and the percent difference from the mean of all sequences prior to outlier corrections.

**Table 3:** TKV measurement variations among five sequences.

			Percent Difference of TKV among Every Pair of Sequences (%) *			
	SD (mL) *	CV (%) *	Mean	SD	Min	Max
Model	86	5.0	6.0	4.0	0.8	12.4
Reader 1 (CZ)	75	4.1	4.8	3.4	0.6	10.0
Reader 2 (AS)	85	4.9	5.9	3.9	0.9	12.1
Reader 3 (HD)	90	5.1	6.1	4.0	0.9	12.6
Reader mean	82	4.6	5.4	3.6	0.8	11.2

* All values are the mean over all exams.

**Table 4:** Summary of data acquisition error identified with 10% difference TKV threshold in 109 exams. * Some exams had more than one error.

	All Sequences (*n* = 545)	MR Exams * (*n* = 109)	Axial T2 (*n* = 109)	Coronal T2 (*n* = 109)	Axial T1 (*n* = 109)	Axial SSFP (*n* = 109)	Coronal SSFP (*n* = 109)
Data Acquisition Problem	Count (%)						
Breathing during 2D scan	35 (6%)	27 (25%)	12 (2%)	19 (4%)	0 (0%)	1 (0%)	3 (1%)
Different breath-hold positions	25 (5%)	21 (19%)	7 (1%)	0 (0%)	13 (2%)	5 (1%)	0 (0%)
Overlapping slices	17 (3%)	12 (11%)	6 (1%)	0 (0%)	7 (1%)	4 (1%)	0 (0%)
Incomplete Kidneys	4 (1%)	4 (4%)	0 (0%)	0 (0%)	4 (1%)	0 (0%)	0 (0%)
Total errors	81 (15%)	49 (45%)	25 (5%)	19 (4%)	24 (4%)	10 (2%)	3 (1%)

## Data Availability

Data generated or analyzed during this study are available from Chenglin Zhu by request subject to institutional review and a data use agreement.

## Supplementary Materials

The following supporting information can be downloaded at: https://www.mdpi.com/article/10.3390/tomography9040107/s1, Supplementary Table S1: Repeatability and reproducibility of MR-based TKV measurements in the literature, Supplementary Table S2: Scanners utilized for training and reproducibility data acquisition, Supplementary Table S3: Differences in TKV between 3D model and mean TKV of three expert observers, Supplementary Figure S1: Scatter Plot of Model Accuracy vs. Inter-reader Variation in TKV Measurements, Supplementary Figure S2: A box plot of the CV in TKV among the three observers per sequence, Supplementary Figure S3: Ten Bland Altman plots of sequence-to-sequence comparisons (using average TKV from three readers) prior to acquisition error screening, Supplementary Figure S4: Histograms of the percent difference in TKV caused by each acquisition issue compared to the reference TKV prior to acquisition error screening, Supplementary Figure S5: Inter-sequence coefficient of variation in TKV of MR exams from seven different scanners.
