# Supplementary material for: Clinical Quality Control of MRI Total Kidney Volume Measurements in Autosomal Dominant Polycystic Kidney Disease
Source: Tomography. 2023 Jul 12;9(4):1341–55. doi: 10.3390/tomography9040107 (PMC10366880; doi:10.3390/tomography9040107)
Supplement: Supplementary file 1 [file tomography-09-00107-s001.zip › tomography-2459473-supplementary.pdf]

**Supplementary Materials**

*Supplementary Table S1. Repeatability and reproducibility of MR-based TKV measurements in the literature.*

| First Author | Year | # of Subjects | # of Readers | Modality | Method | Measurement Variability | |
|---|---|---|---|---|---|---|---|
| | | | | | | Intra-Observer | Inter-Observer |
| Bae [19] | 2000 | 4 | | Axial/Coronal T2 | stereology | PD: 1.3% | |
| Bae [16] | 2009 | 20 | 2 | Coronal T1 pre Gd | stereology | PD: 1.06% | PD: 1.04% |
| | | | | Coronal T1 post Gd | stereology | PD: −0.69% | PD: 2.89% |
| Cohen [20] | 2012 | 17 | 4 | Coronal SSFP | Semi-automated manual contour | CV: 2.4% | CV: 7.1% |
| | | | | Coronal HASTE | Semi-automated manual contour | CV: 2% | CV: 6.9% |
| Spithoven [21] | 2015 | 10 | 4 | Coronal T2 FS | Manual contour | CV: 1.8% | CV: 2.3% |
| Turco [22] | 2015 | 15 | 2 | Coronal T2 FS | Semi-automatic manual contour | | PD: 2.5%** |
| Sharma [12] | 2017 | 15 | 2 | Coronal T1 | Manual | CV: 1.2% | CV: 2.8% |
| Van Gastel [17] | 2018 | 40 | 3 | Coronal T2 | Manual | CV: 0.8% | CV: 2.2% |
| | | | | Coronal T1 | Manual | CV: 1.2% | CV: 1.7% |
| Demoulin [11] | 2021 | 10-22 | 2 | Axial T1 | Manual | CV: 2.4% | CV: 6.7% |

CV = coefficient of variation; PD = absolute percent difference. ** 2.6 ± 1.5% and 2.3 ± 1.4% for right and left kidney, respectively. For studies including multiple readers, the average value of all readers or reader-pairs is reported.

*Supplementary Table S2:* Scanners utilized for training and reproducibility data acquisition.

| **Imaging Center** | **Scanner** | **Field Strength (Tesla)** | **Company** | **Manufacturer Model Name** |
|---|---|---|---|---|
| BKMR | BKMR3C034 | 3 | GE | SIGNA Architect |
| | BKMR3C036 | 3 | GE | SIGNA Architect |
| DWCMR | DWCMR734 | 3 | Siemens | MAGNETOM Vida fit |
| | DWCPTMR733 | 3 | GE | SIGNA PET/MR |
| MR55 | MR55_1 | 1.5 | GE | Signa HDxt |
| | MR55_2 | 1.5 | GE | Signa HDxt |
| NYPWCI | NYPWCI_AMBRA | 1.5 | Siemens | Espree |
| SIMR | SIMR3TXWP418 | 3 | Siemens | MAGNETOM Vida fit |
| YABMR | YABMR15X344 | 1.5 | Siemens | MAGNETOM Sola fit |
| | YABMR30X344 | 3 | Siemens | MAGNETOM Vida fit |
| | YABSIMRX340 | 3 | Siemens | MAGNETOM Vida fit |
| | YABSIPTMRX327 | 3 | Siemens | Biograph_mMR |

*Supplementary Table S3. Differences in TKV between 3D model and mean TKV of 3 expert observers.*

| | **Absolute Difference (mL)** | | | | | **Absolute Percent Difference (%)** | | | | |
|---|---|---|---|---|---|---|---|---|---|---|
| **Sequence** | **min** | **25%** | **median** | **75%** | **max** | **min** | **25%** | **median** | **75%** | **max** |
| AX SSFP | 0.33 | 2.33 | 5.33 | 10.3 | 23 | 0.02 | 0.17 | 0.37 | 0.87 | 5.78 |
| AX T2 | 0 | 1.33 | 3.83 | 9.83 | 132 | 0 | 0.13 | 0.34 | 0.67 | 2.83 |
| AX T1 | 0.33 | 3.67 | 9 | 20.8 | 237 | 0.04 | 0.36 | 0.69 | 1.28 | 29.9 |
| COR SSFP | 0 | 2.75 | 6 | 9.58 | 63.7 | 0 | 0.19 | 0.44 | 1.12 | 4.1 |
| COR T2 | 0.33 | 3.75 | 10.5 | 17.3 | 89 | 0.02 | 0.36 | 0.68 | 1.33 | 5.43 |
| All Sequences | 0 | 2.67 | 7 | 13.7 | 237 | 0 | 0.21 | 0.5 | 1.06 | 29.9 |

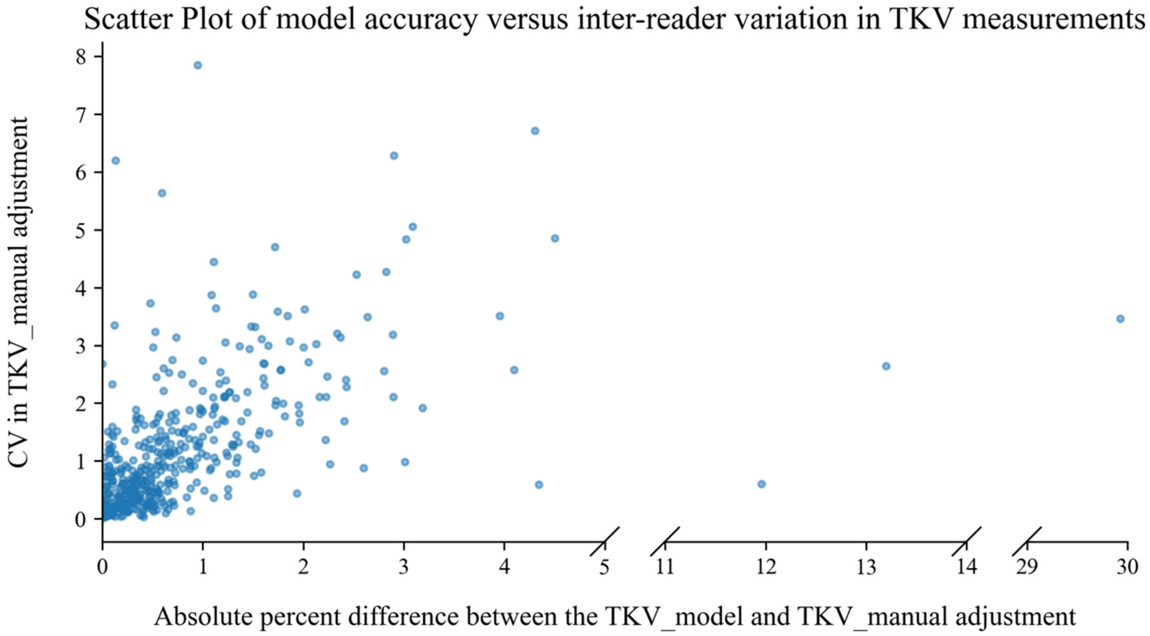


*Supplementary Figure S1: Scatter Plot of Model Accuracy vs. Inter-reader Variation in TKV Measurements.*

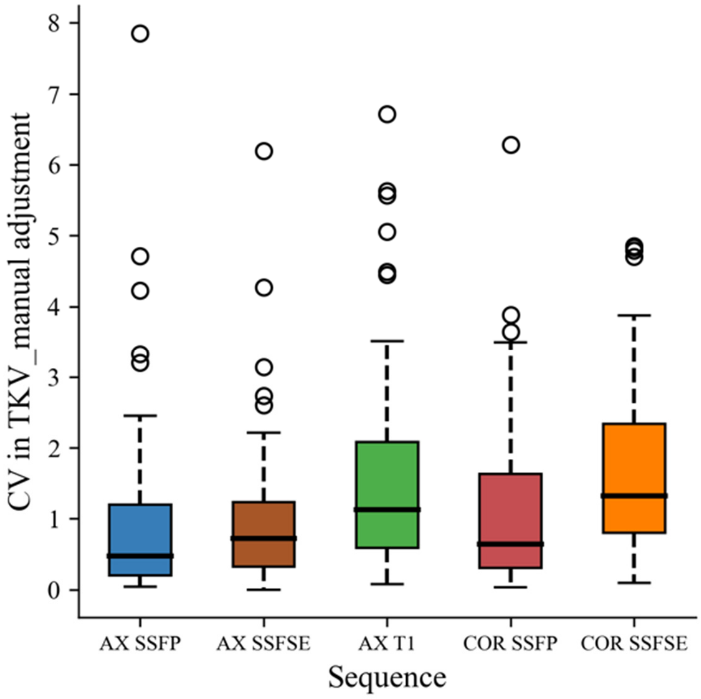


*Supplementary Figure S2: A box plot of the CV in TKV among the 3 observers per sequence.*

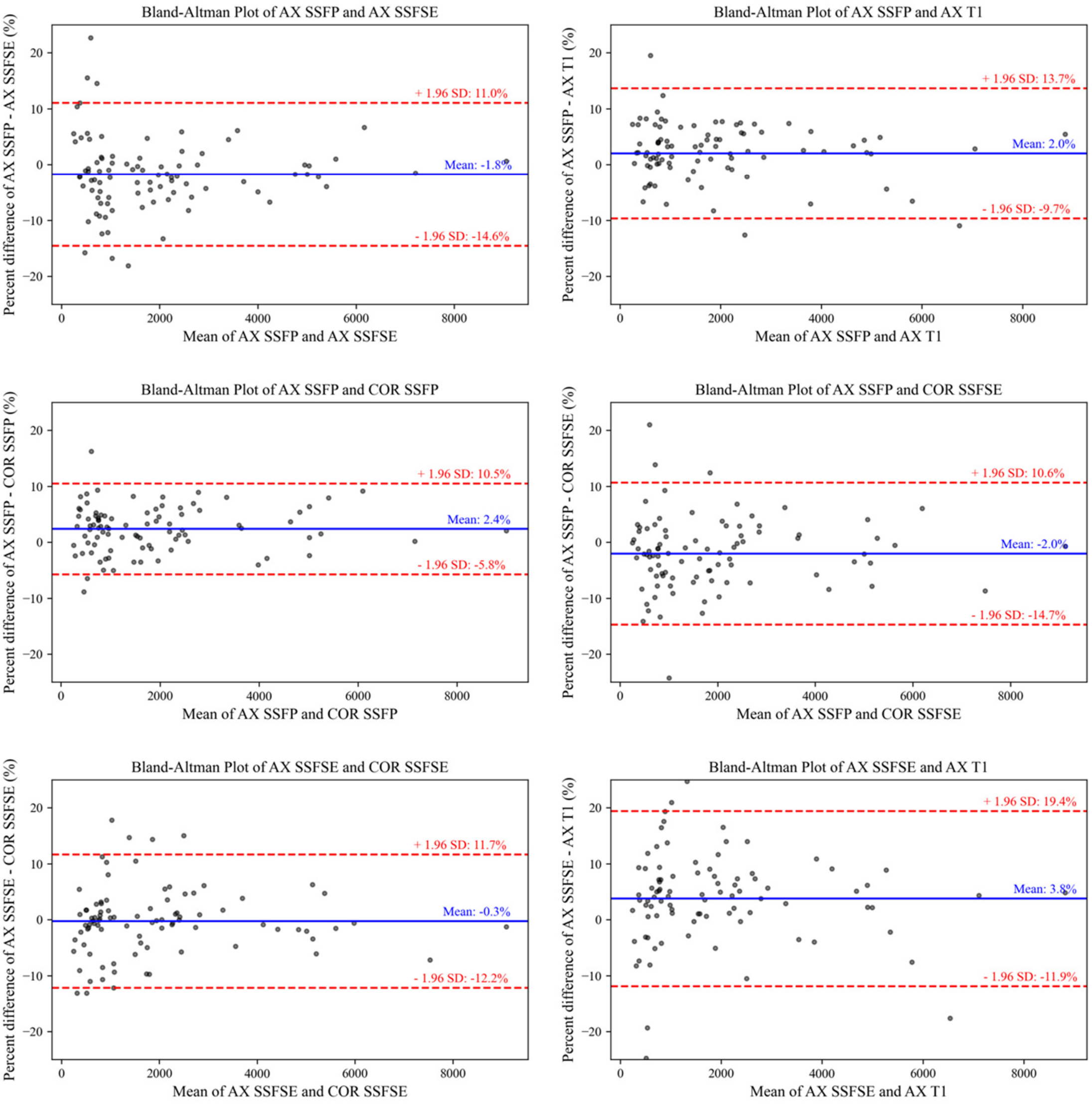
Bland-Altman Plot of AX SSFP and AX SSFSE
Percent difference of AX SSFP - AX SSFSE (%)
Mean of AX SSFP and AX SSFSE
+ 1.96 SD: 11.0%
Mean: -1.8%
- 1.96 SD: -14.6%
Bland-Altman Plot of AX SSFP and AX T1
Percent difference of AX SSFP - AX T1 (%)
Mean of AX SSFP and AX T1
+ 1.96 SD: 13.7%
Mean: 2.0%
- 1.96 SD: -9.7%
Bland-Altman Plot of AX SSFP and COR SSFP
Percent difference of AX SSFP - COR SSFP (%)
Mean of AX SSFP and COR SSFP
+ 1.96 SD: 10.5%
Mean: 2.4%
- 1.96 SD: -5.8%
Bland-Altman Plot of AX SSFP and COR SSFSE
Percent difference of AX SSFP - COR SSFSE (%)
Mean of AX SSFP and COR SSFSE
+ 1.96 SD: 10.6%
Mean: -2.0%
- 1.96 SD: -14.7%
Bland-Altman Plot of AX SSFSE and COR SSFSE
Percent difference of AX SSFSE - COR SSFSE (%)
Mean of AX SSFSE and COR SSFSE
+ 1.96 SD: 11.7%
Mean: -0.3%
- 1.96 SD: -12.2%
Bland-Altman Plot of AX SSFSE and AX T1
Percent difference of AX SSFSE - AX T1 (%)
Mean of AX SSFSE and AX T1
+ 1.96 SD: 19.4%
Mean: 3.8%
- 1.96 SD: -11.9%

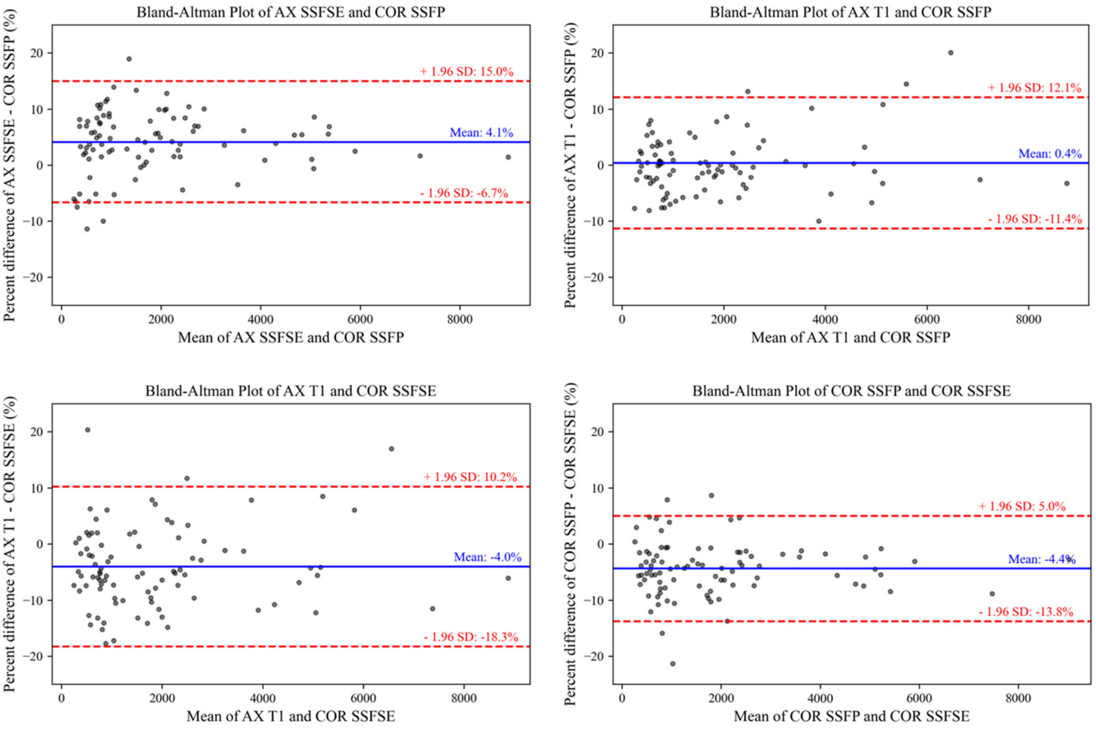


*Supplementary Figure S3：Bland Altman plots of sequence comparisons (using average TKV from three readers).*

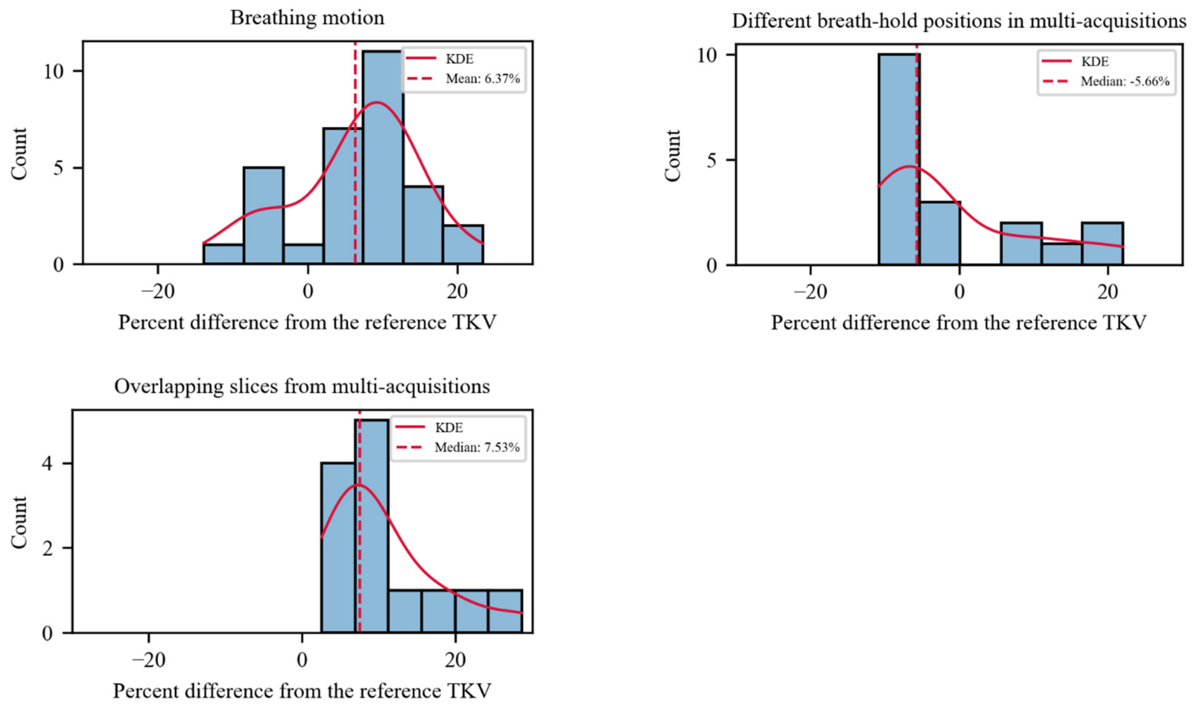


*Supplementary Figure S4: Histograms of the percent difference in TKV caused by each acquisition issue compared to the reference TKV excluding outliers. Breathing motion (top left). Different breath-hold positions in multi-acquisitions (top right). C) Overlapping slices from multi-acquisitions (bottom left). KDE: Kernel Density Estimation.*

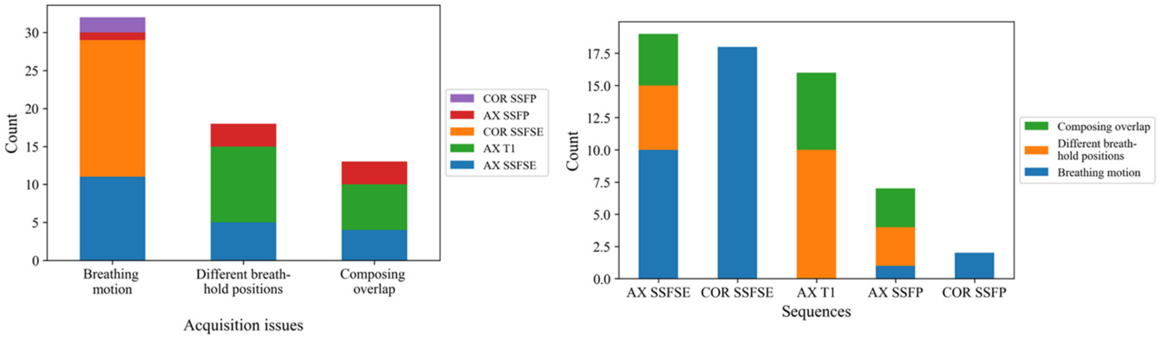


*Supplementary Figure S5: Number of acquisition errors (left) based upon acquisition error type and (right) based upon MRI pulse sequence.*
